# Editorial: Subcellular Compartmentalization of Plant Antioxidants and ROS Generating Systems

**DOI:** 10.3389/fpls.2021.643239

**Published:** 2021-02-18

**Authors:** José M. Palma, Francisco J. Corpas

**Affiliations:** Group of Antioxidants, Free Radicals and Nitric Oxide in Biotechnology, Food and Agriculture, Department of Biochemistry, Cell and Molecular Biology of Plants, Estación Experimental del Zaidín, Consejo Superior de Investigaciones Científcas, Granada, Spain

**Keywords:** chloroplasts, mitochondria, peroxisomes, reactive oxygen species, antioxidants, reactive nitrogen species

The oxygen paradox is a very well-known issue which encounters the necessity of oxygen for life with its toxic potentiality. This simplistic but real fact is molded by the prevailing oxygen concentrations that must be controlled for the optimal functioning of aerobic living beings. In plants, oxygen gains special relevance because, besides being fundamental for respiration, it is also product of the water photolysis occurring at the photosystem II (PSII). This converts chloroplasts into oxygen-enriched organelles and, in fact, this gas is target of the reducing events which take place in this compartment as a consequence of two light reactions at both PSII and PSI. As long as plant colorful organs (mainly green, including leaves, shoots, and fruits) are illuminated, photosynthesis is always operating if NADP^+^ is conveniently available. But, subsidiarily, bypass processes always occur with the concomitant production of reactive oxygen species (ROS), either singlet oxygen (^1^O_2_) at the PSII, and superoxide radicals (${\text{O}}_{2}^{\cdot -}$) at the PSI with subsequent dismutation to hydrogen peroxide (H_2_O_2_) (Foyer and Noctor, 2003; Asada, 2006; Gill and Tuteja, 2010; Corpas et al., 2015).

Under unfavorable stressful conditions, with enhanced ROS production and with chloroplast electron transport overcoming the ferredoxin-NADP reductase (FNR) capacity to reduce NADP^+^ to NADPH for further use in the CO_2_-fixing Calvin-Benson cycle, the oxygenase side of the RuBisCO is stimulated, thus promoting the photorespiratory pathway. This last route involves chloroplasts, peroxisomes and mitochondria. The first peroxisomal enzyme of this route is glycolate oxidase (GOX) which converts glycolate into glyoxylate with the concomitant production of H_2_O_2_. What it started as a stress condition imposed to chloroplast is then translocated to other cell organelles, i.e., peroxisomes, which increase their H_2_O_2_ production by the GOX activity. Additional systems located in other cell compartments like the mitochondrial electron transport chain and the respiratory burst oxidase homolog (Rboh) from plasma membrane are also ROS sources which may contribute to exacerbated ROS levels under stressful conditions. Thus, through the interconnection of organelles in the cell homeostasis, any sudden disturbing situation can be expanded as a snowball to the whole-cell where a set of mechanisms is prone to be activated to control the ROS excess (Asada, 2006; Gill and Tuteja, 2010; Corpas et al., 2015; Czarnocka and Karpinski, 2018; Janku et al., 2019; Kohli et al., 2019).

This ROS-modulating set includes enzymatic and non-enzymatic antioxidants. As plant antioxidative enzymes, superoxide dismutase (SOD), catalase, and the ascorbate-glutathione cycle are the most prominent members, while ascorbate (ASC), glutathione (GSH), carotenoids, α-tocopherol, and polyphenols are the main low-molecular-weight antioxidants. Within these latter ones, ASC is perhaps the most paradigmatic, powerful and versatile antioxidant since it can directly scavenge ROS, either ${\text{O}}_{2}^{\cdot -}$, H_2_O_2_, or hydroxyl radicals (^·^OH), but its effectiveness in controlling H_2_O_2_ levels may increase with the aid of ascorbate peroxidase (APX). ASC also contributes to quench ^1^O_2_ by regenerating α-tocopherol, and in the dynamics of carotenoids (also ^1^O_2_ scavengers), through the xanthophylls' cycle (Gupta et al., 2018). Each organelle contains its own antioxidative equipment, but the participation of ASC and GSH cell-wide grants to these molecules a relevant role in the oxidative homeostasis. ASC is synthesized in the mitochondrial membrane whereas the synthesis of GSH takes place in the cytosol and chloroplasts (Arisi et al., 1997; Zechmann, 2014, 2018; Rodríguez-Ruiz et al., 2017; Jez, 2019; Martí et al.). However, in still little known processes, these compounds migrate to all organelles to exert their antioxidant role, either directly or through the participation of enzymatic partners (Figure 1).

**Figure 1:**
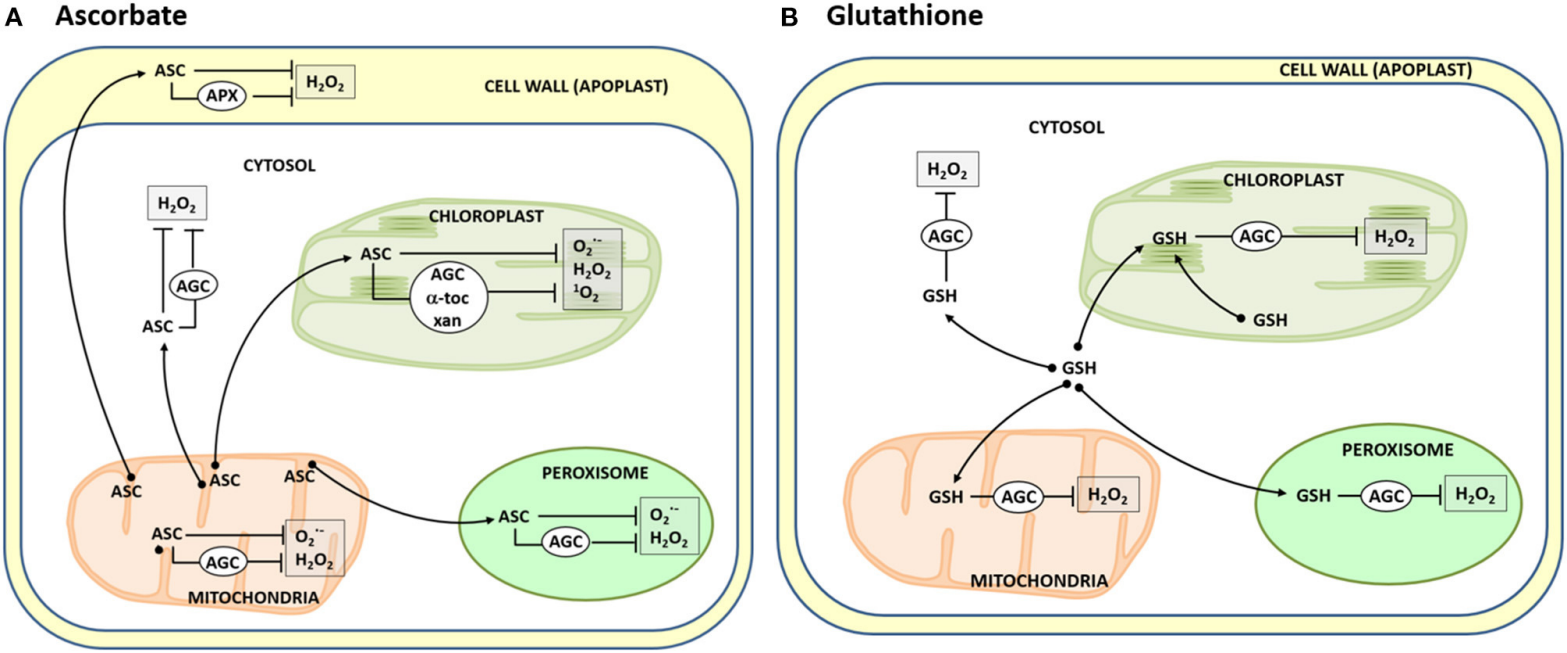
Dynamics of ascorbate **(A)** and glutathione **(B)** in plant cells. Ascorbate (ASC) generated in the mitochondrial membranes [see Rodríguez-Ruiz et al. (2017) and Martí et al.] is driven to the different cell loci where it is used to neutralize diverse ROS [superoxide radicals (${\text{O}}_{2}^{\cdot -}$), hydrogen peroxide (H_2_O_2_), and singlet oxygen (^1^O_2_)] generated in the organelles. This occurs directly or indirectly through either the participation of the ascorbate peroxidase (APX) and the enzymes of the ascorbate-glutathione cycle (AGC), regenerating α-tocopherol (α-toc), or the xanthophylls' cycle (xan). Likewise, glutathione (GSH), synthesized in the cytosol and chloroplasts, is basically used as well in different cell compartments to indirectly scavenge H_2_O_2_ through the AGC.

Accordingly, this Research Topic includes articles focused on the main plant cell organelles which harbor the most active ROS and antioxidant metabolism in the cell: chloroplasts, mitochondria and peroxisomes. Thus, it has been reported that in mulberry (*Morus* ssp.), a chloroplast drought-induced stress protein (MaCDSP32), with thioredoxin nature, is upregulated under drought conditions and appears to confer drought tolerance and ROS homeostasis, what triggers the stress response during seed germination and growth of this plant species (Sun et al.). Salt and osmotic stresses have been also addressed in this volume, and it was found that the exogenous application of the flavonoid naringenine protected bean (*Phaseolus vulgaris*) chloroplast against those stress conditions by regulating the PSII efficiency and the organelle antioxidant machinery (Yildiztugay et al.). The involvement of chloroplasts in plant immunity and disease with the assistance of other organelles such as apoplast, mitochondria, and peroxisomes and their respective ROS-redox systems was reviewed, and the integration between photosynthesis and plant immunity for future food demand was proposed (Kuzniak and Kopczewski). The role of ROS and antioxidant agents from mitochondria and plastids, and the profile of DNA damage in those organelles were also analyzed in different organs during maize development and under dark and light conditions (Tripathi et al.).

The involvement of signaling processes mediated by ROS, reactive nitrogen species (RNS) and H_2_S in the response to biotic and abiotic was also investigated at mitochondrial level in this Research Topic. It was discussed how these chemical species operate in these organelles through diverse post-translational modifications (PTMs) including *S*-oxidation, *S*-glutathionylation and *S*-nitrosation, paying special attention to the thioredoxin/peroxiredoxin system as target and following its response to environmental stress (Martí et al.). *S*-nitrosation and nitration promoted by RNS, and persulfidation mediated by H_2_S are PTMs which take part as strategic tools for the autoregulation of the peroxisomal metabolism to adapt these organelles to their target organ, the developmental stage, and to face external stimuli including pathogens and abiotic agents. These signaling processes, together with the crosstalk among ROS, RNS, reactive sulfur species (RSS), and peroxisomes under the prevailing conditions have been also reviewed here (Corpas et al.).

Additionally, this Research Topic covers glycosylation of antioxidant molecules and phytohormones achieved by UDP-glycosyltransferases with consequences in their cell and histological repartition. By this modification, plants upgrade their adaptation and fitness capacities in a scenario of climate change which can influence the productivity of many crops (Behr et al.). In this context, a synergistic regulation of nitrogen (N) and sulfur (S) on the redox and amino acid balances has been proposed to be a key point to improve the maize sustainable yield and its nutritional quality. Consequently, a balanced N-S stoichiometry promoted enhanced GSH content and photosynthetic rate (Liu et al.).

Overall, this Research Topic provides an updated overview on how the synergy among diverse strategies at subcellular and molecular levels can bring light to many aspects of the ROS and antioxidant cell homeostasis, what conveys the potential repercussion of these promising investigations for the upcoming modern agriculture.

## Author Contributions

All authors listed have made a substantial, direct and intellectual contribution to the work, and approved it for publication.

## Conflict of Interest

The authors declare that the research was conducted in the absence of any commercial or financial relationships that could be construed as a potential conflict of interest.
